# Supplementary material for: Transcripts with high distal heritability mediate genetic effects on complex metabolic traits
Source: Nat Commun. 2025 Jul 1;16:5507. doi: 10.1038/s41467-025-61228-9 (PMC12216720; doi:10.1038/s41467-025-61228-9)
Supplement: Supplementary file 1 — Supplementary Information [file 41467_2025_61228_MOESM1_ESM.pdf]

Supplementary Figures for: Transcripts with high distal heritability mediate genetic effects on complex metabolic traits

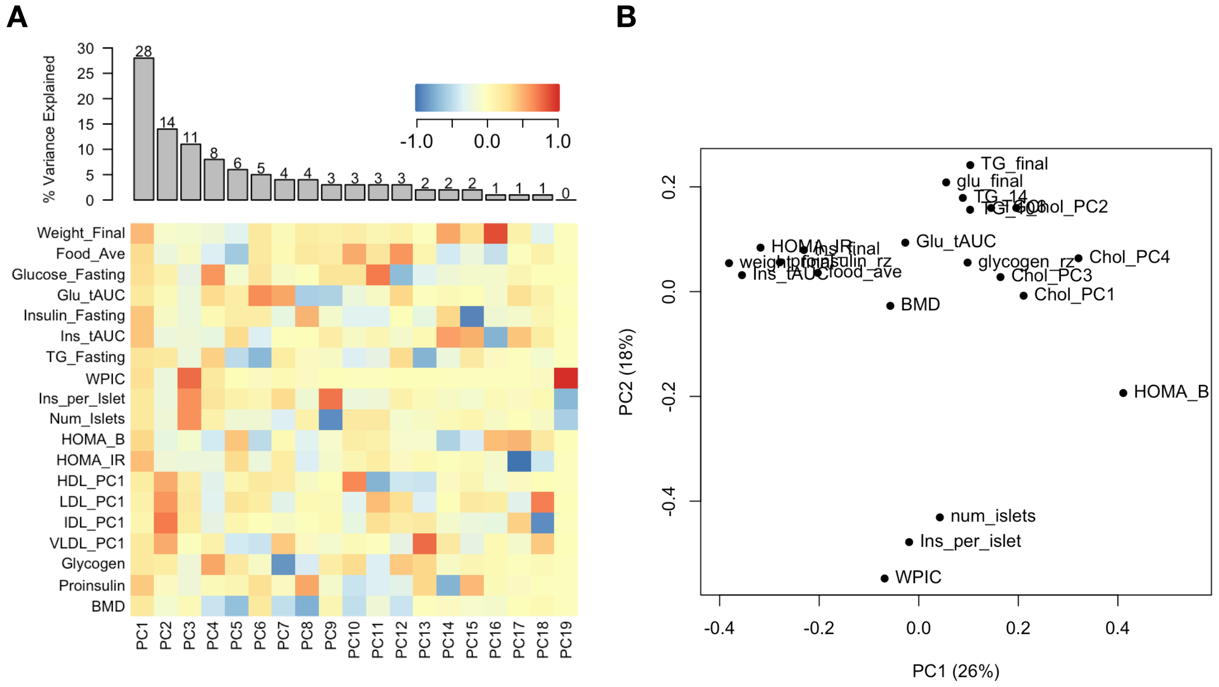

Supplementary Figure 1: Trait matrix decomposition. **A** The heat map shows the loadings of each trait onto each principal component of the trait matrix derived from $n = 471$ DO mice. The bars at the top show the percent variance explained for each principal component. **B** Traits plotted by the first and second principal components of the trait matrix. This view shows clustering of traits into insulin- and weight-related traits, lipid-related traits, and ex-vivo pancreatic measurements. Source data are provided as a Source Data file.

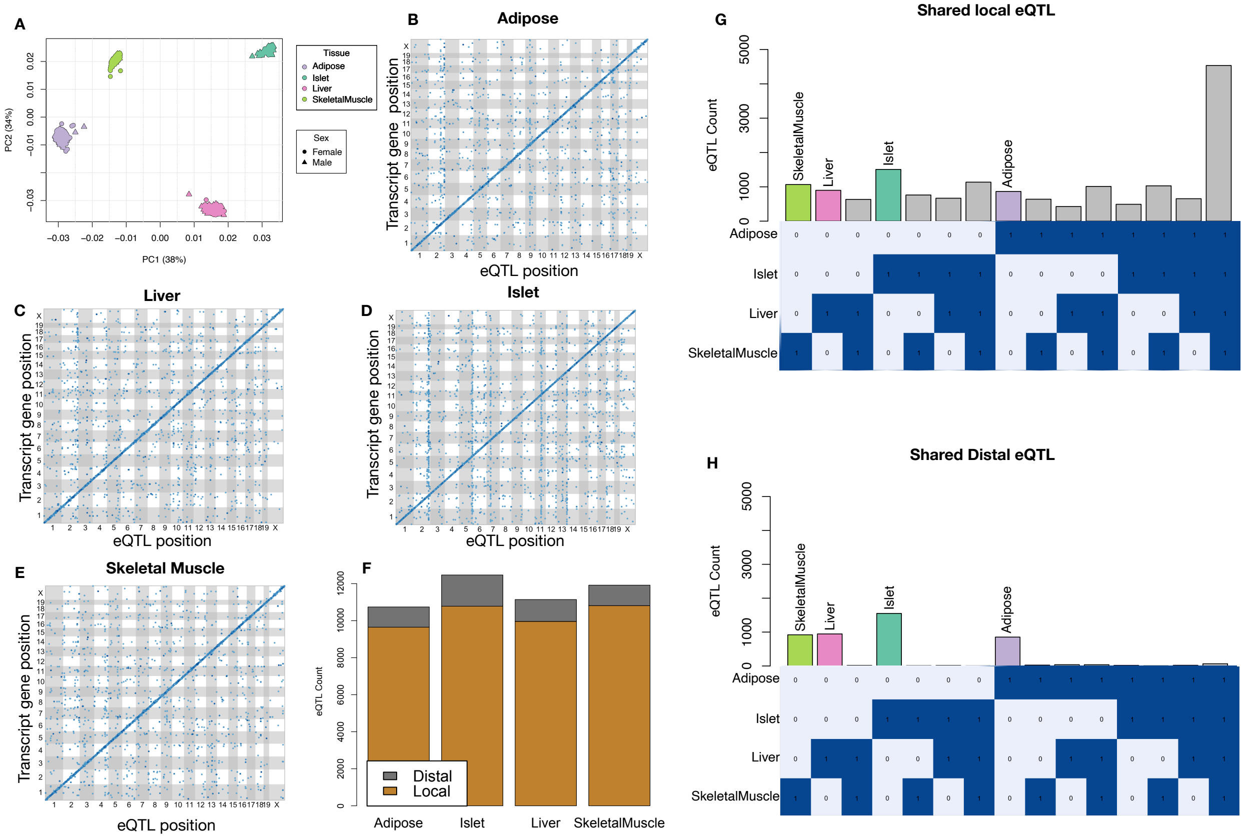

Supplementary Figure 2: Overview of eQTL analysis in DO mice. **A.** RNA seq samples from the four different tissues clustered by tissue. **B.-E.** eQTL maps are shown for (B) adipose tissue ($n = 227$ females, $n = 241$ males), (C) liver ($n = 240$ females, $n = 241$ males), (D) pancreatic islet ($n = 188$ females, $n = 190$ males), and (E) skeletal muscle ($n = 235$ females, $n = 239$ males). The x-axis shows the position of the mapped eQTL, and the y-axis shows the physical position of the gene encoding each mapped transcript. Each dot represents an eQTL with a minimum LOD score of 8, which represents a genome-wide permutation-based threshold of $p < 0.01$. The dots on the diagonal are locally regulated eQTL for which the mapped eQTL is at the within 4Mb of the encoding gene. Dots off the diagonal are distally regulated eQTL for which the mapped eQTL is distant from the gene encoding the transcript. **F.** Comparison of the total number of local and distal eQTL with a minimum LOD score of 8 in each tissue. All tissues have comparable numbers of eQTL. Local eQTLs are much more numerous than distal eQTL. **G.** Counts of transcripts with local eQTL shared across multiple tissues. The majority of local eQTLs were shared across all four tissues. **H.** Counts of transcripts with distal eQTL shared across multiple tissues. The majority of distal eQTL were tissue-specific and not shared across multiple tissues. For both G and H, eQTL for a given transcript were considered shared in two tissues if they were within 4Mb of each other. Colored bars indicate the counts for individual tissues for easy of visualization. Source data are provided as a Source Data file.

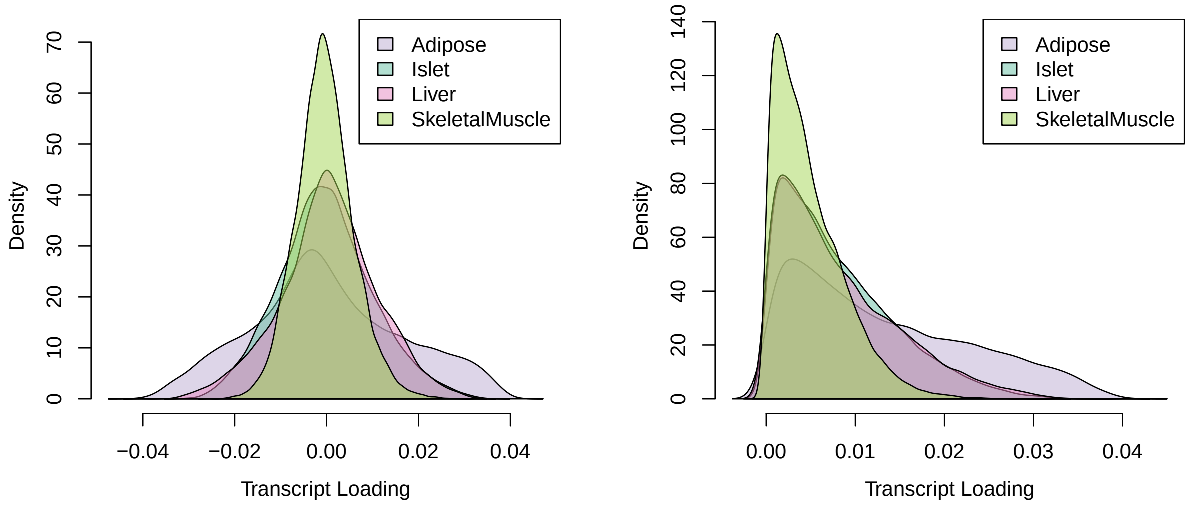

Supplementary Figure 3: Direct comparisons of transcript loadings across tissues also shown in main Figure 4D. **A.** Distributions of transcript loadings are shown as density curves and are differentially colored to indicate tissue. Transcripts in adipose tissue had both the largest positive and negative loadings. **B.** Direct comparison of absolute values of transcript loadings across tissues. Transcripts in adipose tissue had the largest loadings overall, while those in skeletal muscle had the smallest. Source data are provided as a Source Data file. The data shown in this figure are the same as in Fig 4D.

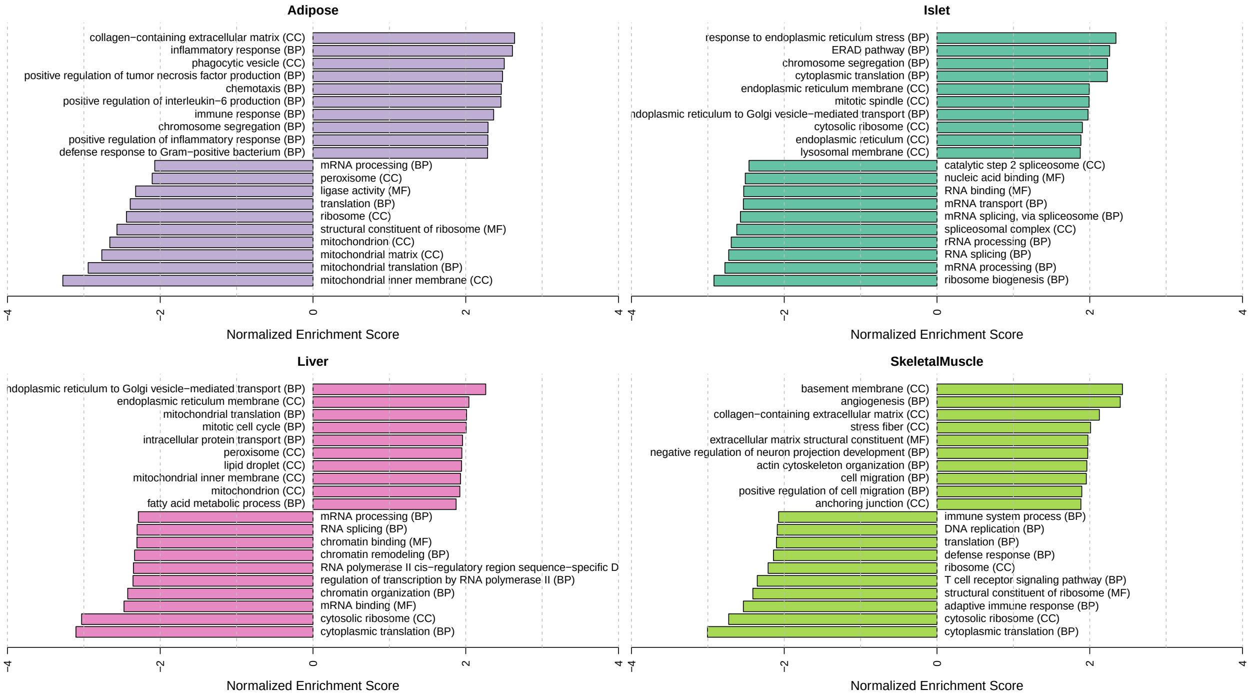

Supplementary Figure 4: Bar plots showing normalized enrichment scores (NES) for GO terms as determined by fast gene score enrichment analysis (fgsea). Only the top 10 positive and top 10 negative scores are shown. Colors indicate tissue. The name beside each bar shows the name of each enriched GO term. The letters in parentheses indicate whether the term is from the biological process ontology (BP), the molecular function ontology (MF), or the cellular compartment ontology (CC). Source data are provided as a Source Data file.

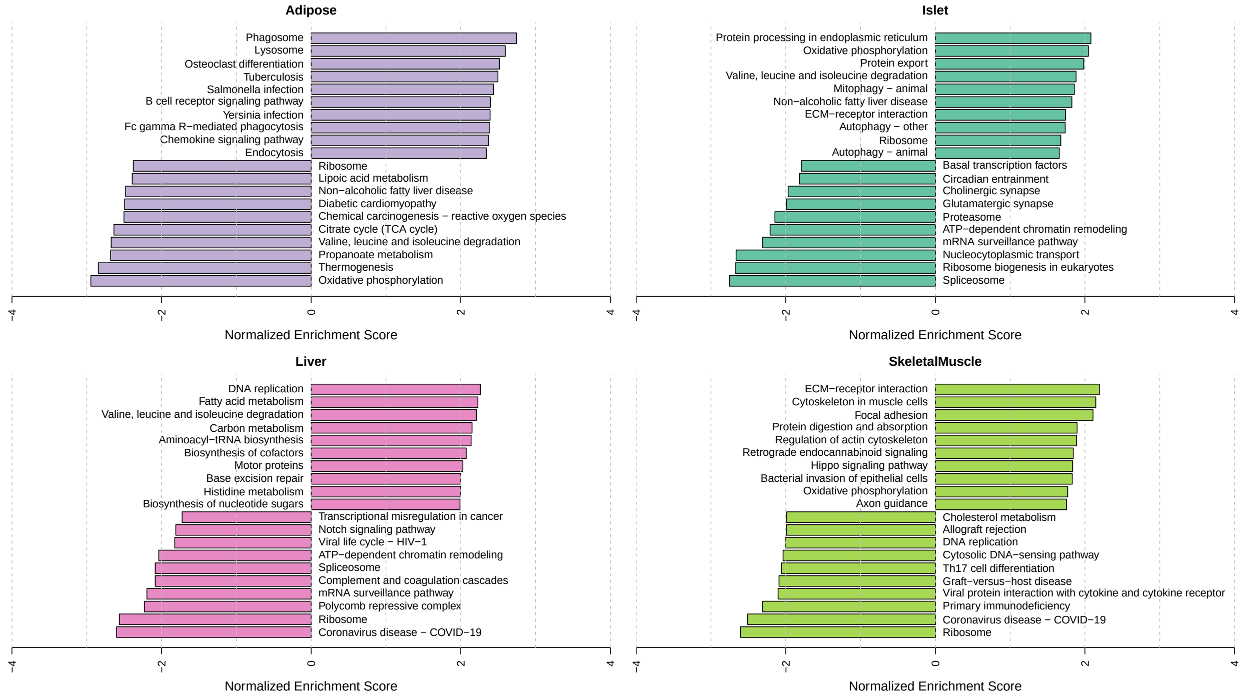

Supplementary Figure 5: Bar plots showing normalized enrichment scores (NES) for KEGG pathways as determined by fast gene score enrichment analysis (fgsea). Only the top 10 positive and top 10 negative scores are shown. Colors indicate tissue. The name beside each bar shows the name of each enriched KEGG pathway. Source data are provided as a Source Data file.

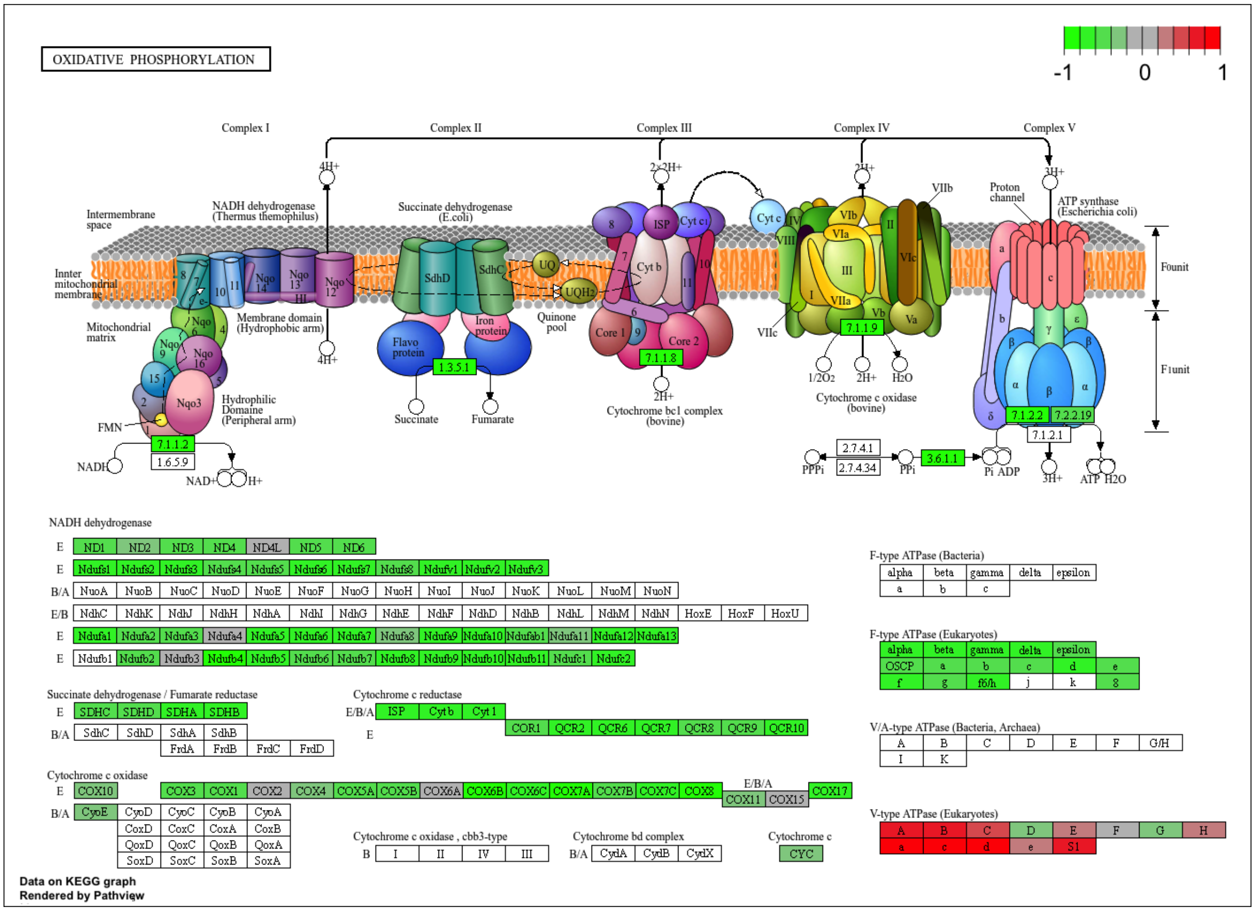

Supplementary Figure 6: The KEGG pathway for oxidative phosphorylation in mice. Each element is colored based on its HDMA loading from adipose tissue scaled to run from -1 to 1. Genes highlighted in green had negative loadings, and those highlighted in red had positive loadings. Almost the entire pathway was strongly negatively loaded indicating that increased expression of genes involved in oxidative phosphorylation was associated with reduced MDI. Source data are provided as a Source Data file.

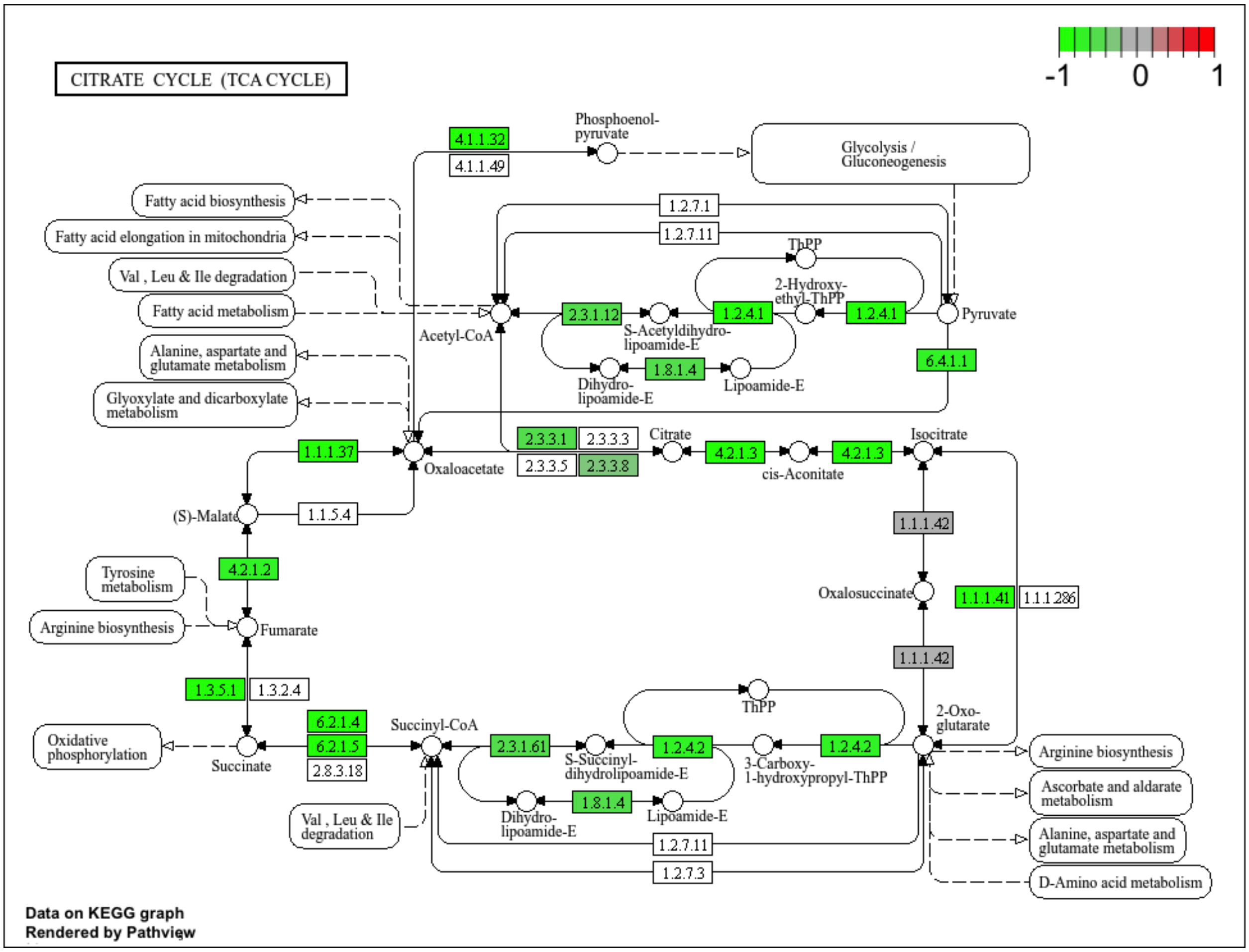

Supplementary Figure 7: The KEGG pathway for the TCA (citric acid) cycle in mice. Each element is colored based on its HDMA loading from adipose tissue scaled to run from -1 to 1. Genes highlighted in green had negative loadings, and those highlighted in red had positive loadings. Many genes in the cycle were strongly negatively loaded indicating that increased expression of genes involved in the TCA cycle was associated with reduced MDI. Source data are provided as a Source Data file.

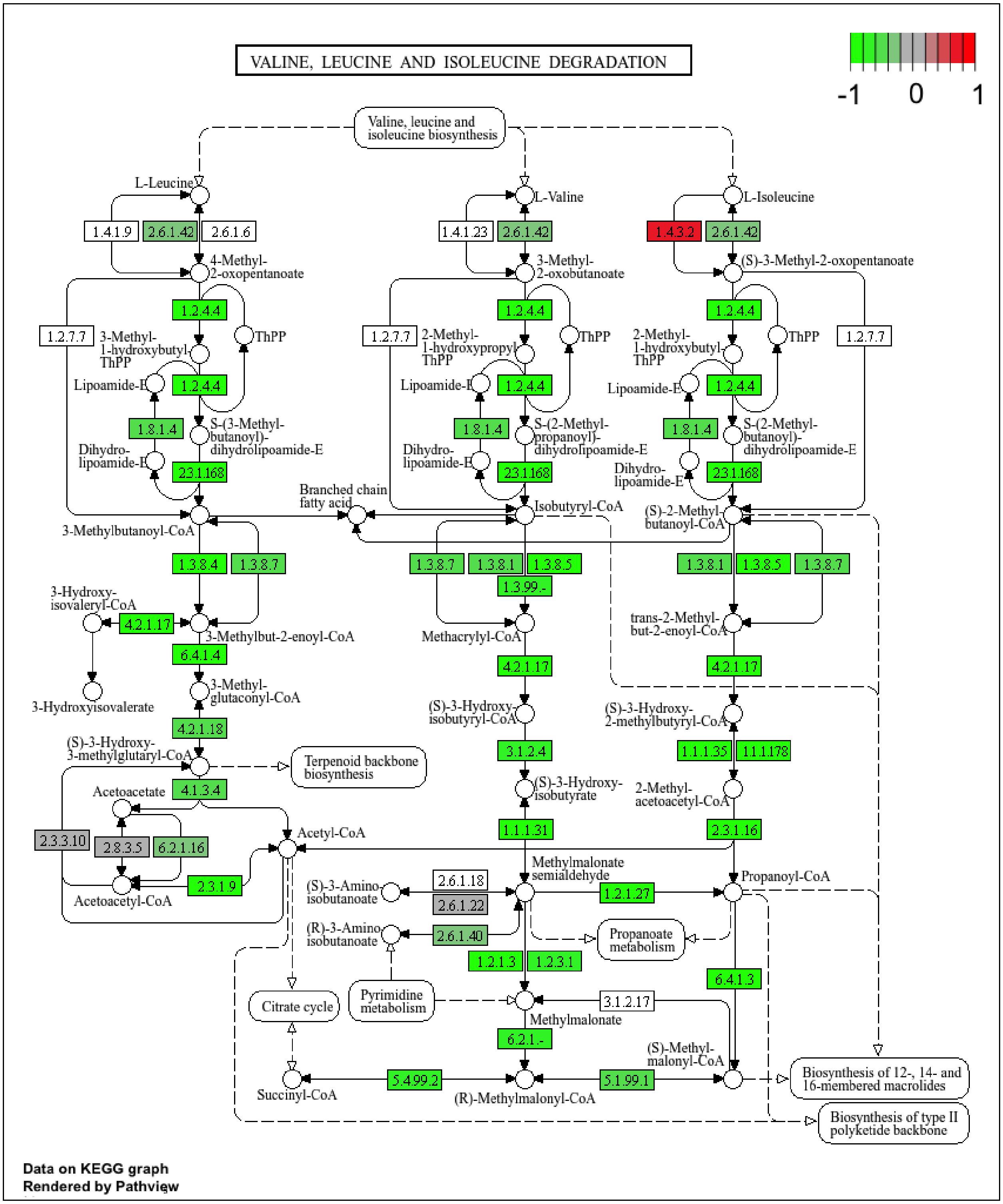

Supplementary Figure 8: The KEGG pathway for branched-chain amino acid degradation in mice. Each element is colored based on its HDMA loading from adipose tissue scaled to run from -1 to 1. Genes highlighted in green had negative loadings, and those highlighted in red had positive loadings. Almost the entire pathway was strongly negatively loaded indicating that increased expression of genes involved in branched-chain amino acid degradation was associated with reduced MDI. Source data are provided as a Source Data file.

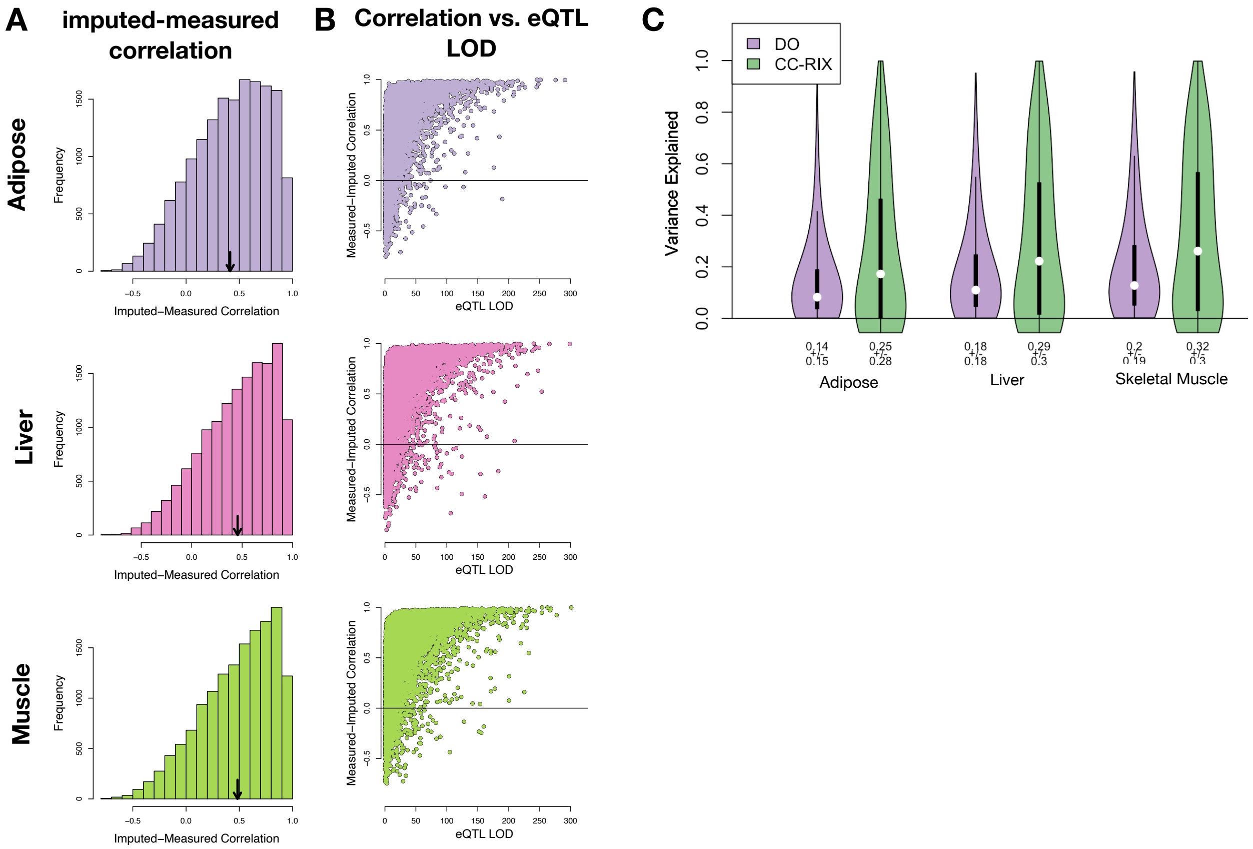

Supplementary Figure 9: Validation of transcript imputation in the CC-RIX. **A.** Distributions of correlations between imputed and measured transcripts in the CC-RIX across tissues distinguished by color. The arrows indicate the mean of each distribution. All distributions were skewed toward positive correlations and had positive means near a Pearson correlation (r) of 0.5. **B.** The relationship between the correlation between measured and imputed expression in the CC-RIX (x-axis) and eQTL LOD score. As expected, imputations are more accurate for transcripts with strong local eQTL. **C.** Variance explained by local genotype in the DO (purple) and CC-RIX (green). Source data are provided as a Source Data file.

cell_iname	pert_type	raw_cs▲	fdr_q_nlog10	set_type	src_set_id
HA1E	TRT_CP	-0.97	15.65	PCL	CP_PROTEIN_SYNTHESIS_INHIBITOR
PC3	TRT_SH.CGS	-0.90	15.65	PATHWAY_SET	BIOCARTA_EIF4_PATHWAY
A375	TRT_CP	-0.87	15.65	MOA_CLASS	RAF_INHIBITOR
HCC515	TRT_CP	-0.84	15.65	PCL	CP_TOPOISOMERASE_INHIBITOR
HEPG2	TRT_SH.CGS	-0.82	15.65	PATHWAY_SET	BIOCARTA_BCR_PATHWAY
PC3	TRT_CP	-0.77	15.65	MOA_CLASS	MTOR_INHIBITOR
HCC515	TRT_CP	-0.76	15.65	PCL	CP_GLUCOCORTICOID_RECEPTOR_AGONIST
HCC515	TRT_CP	-0.76	15.65	MOA_CLASS	GLUCOCORTICOID_RECEPTOR_AGONIST
A375	TRT_CP	-0.72	15.65	MOA_CLASS	MTOR_INHIBITOR
-666	TRT_CP	-0.70	15.65	PCL	CP_PROTEIN_SYNTHESIS_INHIBITOR
-666	TRT_CP	-0.68	15.65	PCL	CP_JAK_INHIBITOR
A549	TRT_CP	-0.67	15.65	PCL	CP_GLUCOCORTICOID_RECEPTOR_AGONIST
A549	TRT_CP	-0.67	15.65	MOA_CLASS	GLUCOCORTICOID_RECEPTOR_AGONIST
-666	TRT_CP	-0.57	15.65	PCL	CP_MTOR_INHIBITOR
-666	TRT_CP	-0.55	15.65	MOA_CLASS	MTOR_INHIBITOR
-666	TRT_CP	-0.55	15.65	PCL	CP_PI3K_INHIBITOR
-666	TRT_CP	0.85	15.65	MOA_CLASS	PKC_ACTIVATOR

Supplementary Figure 10: CMAP results using the adipose tissue composite transcript as an input. Table includes results from all cell types sorted with a $-\log_{10}(q) > 15$. The results are sorted by the correlation of the query to the input with the most negative results at the top. Colored text distinguishes cell and perturbation types to aid visualization. Source data are provided as a Source Data file.

cell_iname	pert_type	raw_cs▲	fdr_q_nlog10	set_type	src_set_id
VCAP	TRT_SH.CGS	-0.99	15.65	PATHWAY_SET	REACTOME_DOWNSTREAM_TCR_SIGNALING
VCAP	TRT_SH.CGS	-0.99	15.65	PATHWAY_SET	REACTOME_NOD1_2_SIGNALING_PATHWAY
A549	TRT_SH.CGS	-0.92	15.65	PATHWAY_SET	BIOCARTA_TNFR1_PATHWAY
VCAP	TRT_SH.CGS	-0.92	15.65	PATHWAY_SET	HALLMARK_WNT_BETA_CATENIN_SIGNALING
HT29	TRT_CP	-0.92	15.65	PCL	CP_TUBULIN_INHIBITOR
-666	TRT_OE	-0.88	15.65	PCL	OE_CELL_CYCLE_INHIBITION
VCAP	TRT_SH.CGS	-0.87	15.65	PATHWAY_SET	REACTOME_P75_NTR_RECEPTOR_MEDIATED_SIGNALLING
HT29	TRT_CP	-0.86	15.65	MOA_CLASS	TUBULIN_INHIBITOR
MCF7	TRT_CP	-0.85	15.65	PCL	CP_TUBULIN_INHIBITOR
-666	TRT_CP	-0.81	15.65	PCL	CP_PROTEASOME_INHIBITOR
-666	TRT_SH.CGS	-0.80	15.65	PATHWAY_SET	REACTOME_DOWNREGULATION_OF_ERBB2_ERBB3_SIGNALING
HCC515	TRT_CP	-0.80	15.65	PCL	CP_GLUCOCORTICOID_RECEPTOR_AGONIST
HCC515	TRT_CP	-0.80	15.65	MOA_CLASS	GLUCOCORTICOID_RECEPTOR_AGONIST
A549	TRT_OE	-0.78	15.65	PATHWAY_SET	REACTOME_RAF_MAP_KINASE_CASCADE
A549	TRT_OE	-0.78	15.65	PATHWAY_SET	PID_RAS_PATHWAY
-666	TRT_SH.CGS	-0.78	15.65	PCL	KD_RIBOSOMAL_40S_SUBUNIT
A549	TRT_OE	-0.76	15.65	PATHWAY_SET	REACTOME_SIGNALLING_TO_P38_VIA_RIT_AND_RIN
A549	TRT_OE	-0.76	15.65	PATHWAY_SET	REACTOME_PROLONGED_ERK_ACTIVATION_EVENTS
A549	TRT_OE	-0.73	15.65	PATHWAY_SET	PID_TCR_RAS_PATHWAY
HA1E	TRT_OE	-0.73	15.65	PATHWAY_SET	REACTOME_SHC_RELATED_EVENTS
HA1E	TRT_OE	-0.71	15.65	PATHWAY_SET	PID_EPHB_FWD_PATHWAY
-666	TRT_CP	-0.70	15.65	MOA_CLASS	GLYCOGEN_SYNTHASE_KINASE_INHIBITOR
HA1E	TRT_OE	-0.70	15.65	PATHWAY_SET	PID_GMCSF_PATHWAY
A549	TRT_OE	-0.69	15.65	PATHWAY_SET	REACTOME_SIGNALLING_TO_ERKS
-666	TRT_LIG	-0.69	15.65	PATHWAY_SET	PID_ERBB_NETWORK_PATHWAY
-666	TRT_CP	-0.67	15.65	MOA_CLASS	PROTEASOME_INHIBITOR
-666	TRT_CP	-0.66	15.65	PCL	CP_GLYCOGEN_SYNTHASE_KINASE_INHIBITOR
-666	TRT_CP	0.73	15.65	MOA_CLASS	MTOR_INHIBITOR

Supplementary Figure 11: CMAP results using the pancreatic islet tissue composite transcript as an input. Table includes results from all cell types sorted with a $-log_{10}(q) > 15$. The results are sorted by the correlation of the query to the input with the most negative results at the top. Colored text distinguishes cell and perturbation types to aid visualization. Source data are provided as a Source Data file.

cell_iname	pert_type	raw_cs ▲	fdr_q_nlog10	set_type	src_set_id
ASC	TRT_CP	-0.94	0.79	PCL	CP_PARP_INHIBITOR
ASC	TRT_CP	-0.94	0.79	MOA_CLASS	PROTEIN_TYROSINE_KINASE_INHIBITOR
ASC	TRT_CP	-0.84	0.45	MOA_CLASS	BTK_INHIBITOR
ASC	TRT_CP	-0.81	0.39	MOA_CLASS	LEUCINE_RICH_REPEAT_KINASE_INHIBITOR
ASC	TRT_CP	-0.81	0.79	PCL	CP_HSP_INHIBITOR
ASC	TRT_CP	-0.80	0.93	PCL	CP_EGFR_INHIBITOR
ASC	TRT_CP	-0.79	0.32	MOA_CLASS	T-TYPE_CALCIUM_CHANNEL_BLOCKER
ASC	TRT_CP	-0.79	1.09	PCL	CP_MTOR_INHIBITOR
ASC	TRT_CP	-0.76	0.97	PCL	CP_PI3K_INHIBITOR
ASC	TRT_CP	-0.75	0.20	MOA_CLASS	HISTONE_DEMETHYLASE_INHIBITOR
ASC	TRT_CP	-0.74	0.42	PCL	CP_IKK_INHIBITOR
ASC	TRT_CP	-0.74	0.83	PCL	CP_AURORA_KINASE_INHIBITOR
ASC	TRT_CP	-0.74	0.17	PCL	CP_LEUCINE_RICH_REPEAT_KINASE_INHIBITOR
ASC	TRT_CP	-0.72	0.36	PCL	CP_BROMODOMAIN_INHIBITOR
ASC	TRT_CP	-0.71	1.09	MOA_CLASS	TYROSINE_KINASE_INHIBITOR
ASC	TRT_CP	-0.70	0.82	PCL	CP_PROTEIN_SYNTHESIS_INHIBITOR
ASC	TRT_CP	-0.67	0.69	PCL	CP_SRC_INHIBITOR
ASC	TRT_CP	-0.67	0.81	MOA_CLASS	AURORA_KINASE_INHIBITOR
ASC	TRT_CP	-0.65	0.89	MOA_CLASS	FLT3_INHIBITOR
ASC	TRT_CP	-0.62	0.40	MOA_CLASS	FGFR_INHIBITOR
ASC	TRT_CP	-0.59	0.66	MOA_CLASS	MEK_INHIBITOR
ASC	TRT_CP	-0.59	0.13	MOA_CLASS	SYK_INHIBITOR
ASC	TRT_CP	-0.58	0.01	PCL	CP_PKC_INHIBITOR
ASC	TRT_CP	-0.58	0.65	PCL	CP_HDAC_INHIBITOR
ASC	TRT_CP	-0.58	0.65	PCL	CP_ATPASE_INHIBITOR
ASC	TRT_CP	-0.53	0.09	PCL	CP_FLT3_INHIBITOR
ASC	TRT_CP	-0.53	0.42	PCL	CP_P38_MAPK_INHIBITOR
ASC	TRT_CP	-0.53	0.22	MOA_CLASS	IKK_INHIBITOR
ASC	TRT_CP	-0.52	0.58	PCL	CP_VEGFR_INHIBITOR
ASC	TRT_CP	-0.51	-0.00	PCL	CP_T_TYPE_CALCIUM_CHANNEL_BLOCKER

Supplementary Figure 12: CMAP results using the adipose tissue composite transcript as an input. Table includes the top 30 results derived only from normal adipocytes (ASC) regardless of significance. The results are sorted by the correlation of the query to the input with the most negative results at the top. Colored text distinguishes cell and perturbation types to aid visualization. Source data are provided as a Source Data file.

cell_iname	pert_type	raw_cs ▲	fdr_q_nlog10	set_type	src_set_id
YAPC	TRT_CP	-1.00	0.67	MOA_CLASS	ABL_KINASE_INHIBITOR
YAPC	TRT_CP	-0.99	0.66	PCL	CP_CDK_INHIBITOR
YAPC	TRT_CP	-0.97	1.41	PCL	CP_TOPOISOMERASE_INHIBITOR
YAPC	TRT_CP	-0.95	0.70	MOA_CLASS	THYMIDYLATE_SYNTHASE_INHIBITOR
YAPC	TRT_CP	-0.95	0.62	MOA_CLASS	ADRENERGIC_INHIBITOR
YAPC	TRT_CP	-0.94	0.50	MOA_CLASS	BENZODIAZEPINE_RECEPTOR_ANTAGONIST
YAPC	TRT_CP	-0.89	0.63	PCL	CP_RIBONUCLEOTIDE_REDUCTASE_INHIBITOR
YAPC	TRT_CP	-0.88	0.52	MOA_CLASS	VASOPRESSIN_RECEPTOR_ANTAGONIST
YAPC	TRT_CP	-0.85	0.63	MOA_CLASS	ANGIOTENSIN_RECEPTOR_ANTAGONIST
YAPC	TRT_CP	-0.85	0.33	PCL	CP_CANNABINOID_RECEPTOR_AGONIST
YAPC	TRT_CP	-0.84	0.30	PCL	CP_RETINOID_RECEPTOR_AGONIST
YAPC	TRT_CP	-0.83	1.19	MOA_CLASS	NFKB_PATHWAY_INHIBITOR
YAPC	TRT_CP	-0.83	0.54	MOA_CLASS	DNA_ALKYLATING_DRUG
YAPC	TRT_CP	-0.80	0.50	MOA_CLASS	CHOLESTEROL_INHIBITOR
YAPC	TRT_CP	-0.79	0.15	MOA_CLASS	SULFONYLUREA
YAPC	TRT_CP	-0.78	0.52	MOA_CLASS	HIV_INTEGRASE_INHIBITOR
YAPC	TRT_CP	-0.78	0.13	MOA_CLASS	LEUKOTRIENE_INHIBITOR
YAPC	TRT_CP	-0.78	0.45	PCL	CP_PPAR_RECEPTOR_AGONIST
YAPC	TRT_CP	-0.78	0.54	MOA_CLASS	INSULIN_SENSITIZER
YAPC	TRT_CP	-0.77	0.51	MOA_CLASS	ESTROGEN_RECEPTOR_ANTAGONIST
YAPC	TRT_CP	-0.77	0.76	MOA_CLASS	DNA_SYNTHESIS_INHIBITOR
YAPC	TRT_XPR	-0.77	0.67	PATHWAY_SET	BIOCARTA_PARKIN_PATHWAY
YAPC	TRT_CP	-0.77	0.51	PCL	CP_VEGFR_INHIBITOR
YAPC	TRT_CP	-0.75	0.39	MOA_CLASS	RNA_SYNTHESIS_INHIBITOR
YAPC	TRT_CP	-0.72	0.60	MOA_CLASS	BCR-ABL_KINASE_INHIBITOR
YAPC	TRT_XPR	-0.71	0.66	PATHWAY_SET	BIOCARTA_EIF_PATHWAY
YAPC	TRT_XPR	-0.69	0.54	PATHWAY_SET	PID_CIRCADIAN_PATHWAY
YAPC	TRT_CP	-0.68	0.77	MOA_CLASS	TOPOISOMERASE_INHIBITOR
YAPC	TRT_XPR	-0.64	0.49	PATHWAY_SET	BIOCARTA_CBL_PATHWAY
YAPC	TRT_CP	-0.64	0.53	MOA_CLASS	TUBULIN_INHIBITOR

Supplementary Figure 13: CMAP results using the pancreatic islet composite transcript as an input. Table includes the top 30 results derived only from YAPC cells, which are derived from pancreatic carcinoma cells. Results are shown regardless of significance and are sorted by the correlation of the query to the input with the most negative results at the top. Colored text distinguishes cell and perturbation types to aid visualization. Source data are provided as a Source Data file.

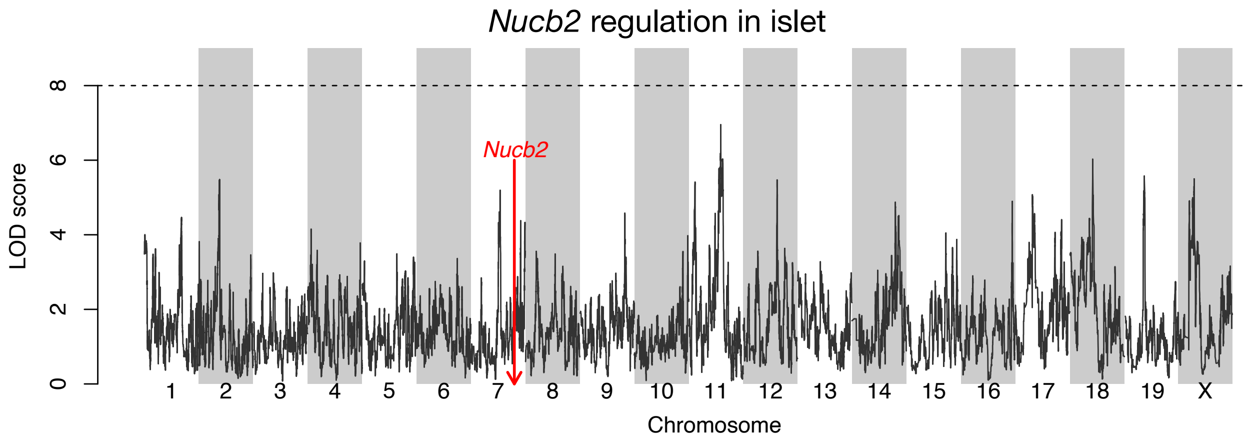

Supplementary Figure 14: Regulation of *Nucb2* expression in islet. *Nucb2* is encoded on mouse chromosome 7 at 116.5 Mb (red arrow). Expression levels of *Nucb2* in islets was 69%. This LOD score trace shows that there is no local eQTL at the position of the gene, nor any strong distal eQTLs. The horizontal dashed line shows the genome-wide FDR threshold of 0.01. Source data are provided as a Source Data file.
