# Supplementary material for: Transcripts with high distal heritability mediate genetic effects on complex metabolic traits
Source: Nat Commun. 2025 Jul 1;16:5507. doi: 10.1038/s41467-025-61228-9 (PMC12216720; doi:10.1038/s41467-025-61228-9)
Supplement: Supplementary file 2 — Description of Additional Supplementary Files [file 41467_2025_61228_MOESM2_ESM.pdf]

Description of Additional Supplementary Files

Supplementary Data 1: Table of gene names, loadings, and cluster identity for genes in Figure 6 - tissues of action

Supplementary Data 2: Table of CC-RIX strains and the numbers of animals in each
